# The use of assistive technology in shoulder exercise rehabilitation – a qualitative study of acceptability within a pilot project

**DOI:** 10.1186/s12891-018-2042-6

**Published:** 2018-05-02

**Authors:** Anthony W. Gilbert, Iva Hauptmannova, Anju Jaggi

**Affiliations:** Royal National Orthopaedic Hospital, Stanmore, UK

**Keywords:** Shoulder, Orthopaedics, Rehabilitation, Technology

## Abstract

**Background:**

Painful shoulders pose a substantial socioeconomic burden accounting for 2.4% of all primary care consultations in the UK. There is a strong evidence to indicate that the majority of this shoulder pain can be managed successfully with exercise based treatments and that common surgical procedures provide no extra benefit. Patient adherence and engagement is cited as an important factor in gaining positive outcomes.

The MUJO System has been designed to help target the rehabilitation of the rotator cuff muscles which are commonly recommended for the management of shoulder pain. The purpose of this qualitative study was to evaluate the acceptability of the MUJO System amongst clinicians and patients.

**Methods:**

A qualitative study was undertaken to look at the usability of the MUJO System both from clinicians’ and patients’ perspectives. Patients with shoulder problems were identified by an experienced physiotherapist using the study eligibility criteria. and invited to participate. Semi-structured interviews were performed with patients and clinicians to explore factors surrounding its acceptability and feasibility of use. The study was designed using Normalisation Process Theory as a theoretical basis for the inquiry.

**Results:**

Seven physiotherapists and ten patients were interviewed in the study. The Internal and External Devices were seen as having the potential to rehabilitate the rotator cuff however it posed limitations towards more functional based exercises. Patients and clinicians found the visual feedback from the Patient App enhanced the rehabilitation experience. The Internal and External Devices were acceptable to all for rehabilitation providing the devices were available for use by the patients in the community.

**Conclusion:**

Patients and clinicians found the MUJO System acceptable as a modality to perform shoulder exercises. For the MUJO System to be taken up as a routine part of clinical practice patients need to be able to access the devices in the community. For the MUJO System to be taken up in clinical practice it needs to be workable within the context of the treatment pathway and not interfere with standard processes.

**Electronic supplementary material:**

The online version of this article (10.1186/s12891-018-2042-6) contains supplementary material, which is available to authorized users.

## Background

Painful shoulders pose a substantial socioeconomic burden accounting for 2.4% of all primary care consultations in the UK [1]. Subacromial pain accounts for up to 70% of all shoulder problems and can impact on work and household tasks [2].

**Fig. 1:**
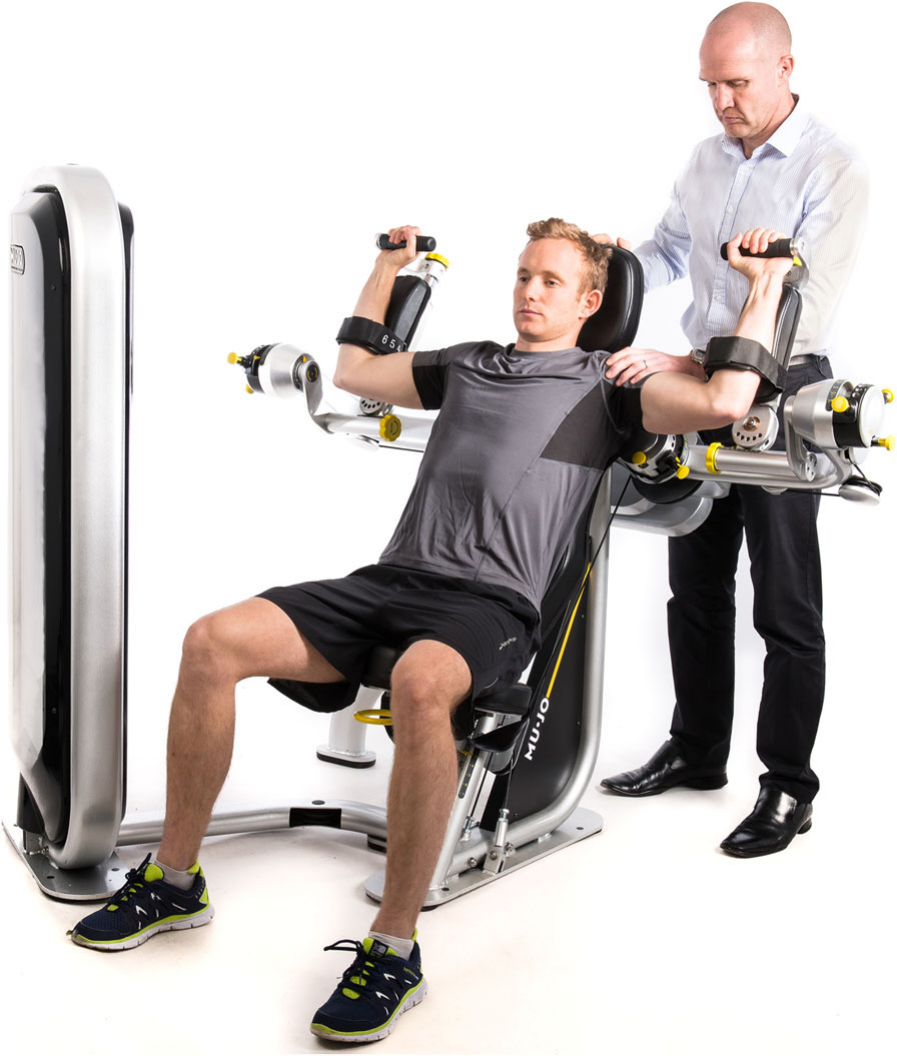
Internal Device [6]. This is a model of a patient using the internal device

**Fig. 2:**
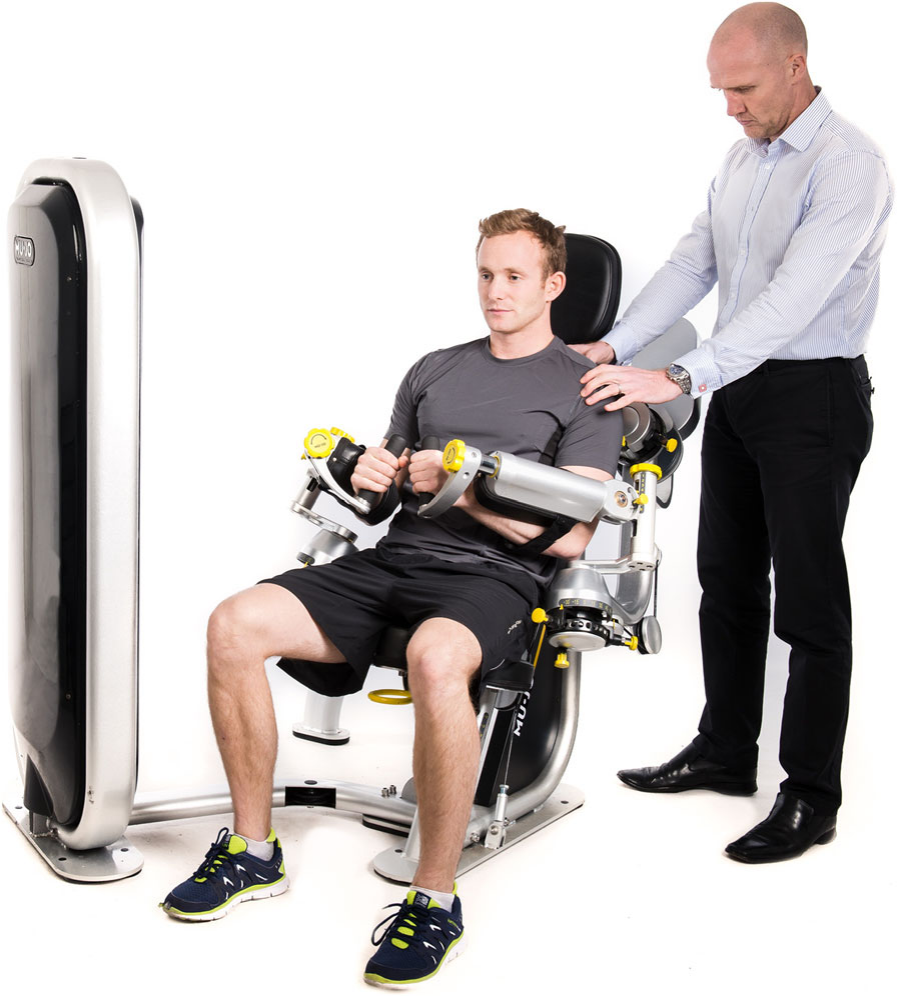
External Device [6]. This is a model of a patient using the external device

There is growing evidence that exercise therapy targeting the rotator cuff muscles can be very effective in managing shoulder pain [3]. Patients are usually seen by a physiotherapist to instruct them on appropriate exercises which they are encouraged to do independently as part of a home exercise program [4]. Undertaking such exercises incorrectly may exacerbate the injury and further delay patient’s recovery. Compliance can also be an issue and adherent patients may have better treatment outcomes than non-adherent patients [5]. Equipment that provides real time feedback to the patient on their level of performance in a controlled environment may contribute to better outcomes. The Multiple Joint (MUJO) System [6] is the first rehabilitation device to train bi-articular muscles or multiple axial joints in a single exercise on one machine. The device was manufactured by MUJO Mechanics in the United Kingdom. The Internal and External Devices allow multiple movement paths to be performed within defined boundaries, to target specific body parts over the full range of motion in a single exercise. The Internal Device (See Fig. 1) provides resistance to internal rotation and the External Device (See Fig. 2) provides resistance to external rotation. The Internal and External Devices use built in sensors to collect joint range angle. All real time movement data is displayed on an accompanied Patient App, displayed on an iPad, connected to the device. This data is collected and stored on a secure cloud database. The Physiotherapy Portal allows the physiotherapist to assess the patient and establish suitable exercise prescriptions. The Patient App has videos and instructions on how to set up the machine based on the prescription set by the physiotherapist.

Several large scale studies, such as the Whole Systems Demonstrator [7], have claimed that if delivered properly, telehealth can reduce mortality, reduce the need for admissions to hospital, lower the number of bed days spent in hospital and reduce the number of Accident and Emergency admissions. The Department of Health in the United Kingdom set up the *‘three million lives*’ campaign [8] with a view to benefit three million people with long term conditions or social needs as it is recognised that technology can positively contribute towards this. However, even in those situations where there is good evidence supporting the uptake of technology in clinical practice, the actual uptake of this technology is low [9]. Systematic reviews have identified considerations for implementation which include whether or not satisfaction is as high for clinicians as well as patients [10], consideration of how the use of technology impacts on roles and responsibilities [11] and healthcare routines [12] and whether or not the intervention limits the possibilities for relationships with professionals and peers [12].

The aim of this study was to determine whether or not the MUJO System was acceptable to patients with shoulder dysfunction and their rehabilitation professionals. We used qualitative methodology to explore the underlying reasons behind the MUJO System’s acceptability [13] and this work was informed by Normalisation Process Theory (NPT) [14]. NPT focuses on the things that participants using the MUJO System must *do* – the work of being a patient or rehabilitation professional – and considers how changes influence the MUJO’s acceptability and feasibility. In this study acceptability is determined as the actual use of the machine and the reasons why or why not participants chose to use it. This research was undertaken during the early stages of testing the MUJO System at the host organisation, a tertiary orthopaedic hospital based in North London (United Kingdom), between March and July 2016.

## Methods

All patients over the age of 18 years attending the host institution (North London, UK) for shoulder rehabilitation were invited into the study provided they met the eligibility criteria. Clinicians were asked to identify patients using the MUJO System. The research physiotherapist then approached patients to invite them into the study. Clinicians were invited to participate in the study providing they had experience of treating patients using the MUJO System. Local Research and Development and research ethics approval were obtained from the London Fulham Research Ethics Committee (15/LO/1683) and written and informed consent was provided by all participants prior to interview. All data was link anonymised.

### Patients inclusion criteria

Patients attended the Royal National Orthopaedic Hospital for shoulder rehabilitation

- Patient remains on the Royal National Orthopaedic Hospital shoulder rehabilitation pathway
- Therapists believe the patient will not be disadvantaged by entering the study
- Patient / clinician available for qualitative interview
- Patients able to communicate in English or languages covered by the host institution interpreter service

### Clinicians inclusion criteria

- Physiotherapist employed by the host institution
- Inducted by MUJO staff into the use of the devices
- Experience of rehabilitating patients using the devices

A single interview was conducted with each study participant (both patients and clinicians) within the Therapies Department of the host institution. Patients undertook the interview upon conclusion of their rehabilitation. Clinicians undertook an interview at the end of the study period. Interviews were conducted by the research physiotherapist (AG), (male, MRes research qualification with qualitative research training and experience) at the host institution and all interviews consisted of the interviewer and participant only. Each interview was audio recorded. The same interview schedule, developed in accordance with NPT [14] was used for all interviews and can be viewed in Additional file 1: Appendix.

### Data analysis

Interviews were transcribed verbatim and entered into a computer assisted qualitative data analysis software programme (QSR NVIVO software – version 11). A Directed Content Analysis [15] was undertaken to organise the qualitative data according to the four constructs of Normalisation Process Theory (NPT) [14]. Data was coded by the research physiotherapist and the results of this study are presented in accordance with the constructs of NPT:

1. Capability – possibilities presented by the MUJO System
2. Capacity – social structural resources available to patients and clinicians
3. Potential – social cognitive resources available to patients and clinicians
4. Contribution – what patients and clinicians do to implement the MUJO System in clinical practice

## Results

Eleven patients and seven physiotherapists, all of whom had experience treating patients using the MUJO System, were invited to join the study and all agreed to do so. During the study period, one patient became unavailable for interview and was removed from the study. Of the ten patients who remained in the study the median age was 38.5 years (19–54), there were five females and the median length of all the patient and clinician interviews was 18.35 min (13.3–31.5). Shoulder pathologies in this group included instability (*n* = 6) and rotator cuff related pain (*n* = 4). Patient demographics are available to view in the Additional file 2. All clinicians were experienced Physiotherapists with more than ten years’ experience and all had sub-specialised in shoulder rehabilitation. Although ten patients and seven physiotherapists were interviewed no new data was identified beyond patient interview 8 and clinician interview 6. No further patients and clinicians were recruited as data saturation appeared to have been achieved. This qualitative study was undertaken in the early stages of the clinical pilot of the MUJO System.

The research physiotherapist had worked in the host institution’s Therapies Department for five years at the time of the study was known to all clinicians prior to the establishment of the study. The research physiotherapist had no prior relationship with the patients and was employed to coordinate the study.

### Capability – Possibilities presented by the MUJO system

Patients and clinicians reported the MUJO System’s benefits. The use of the Internal Device was thought to preferentially target the anterior muscles of the rotator cuff and the External Device to target the posterior muscles of the rotator cuff. Whilst specifically exercising these muscle groups using the machines the physiotherapists were able to observe real time feedback from the Patient App to assess movement control and strength:

*‘The good thing is we can get the cuff and the deltoid working, so in terms of isolating and assessing these patients, I think it’s been quite useful. I use it a lot’* [C4 = Clinician 4]

The MUJO System was seen as having the potential to have an important role in the management of rotator cuff disorders across primary, secondary and tertiary care. Several clinicians discussed the potential usefulness of housing the device in the community to facilitate shoulder rehabilitation:

*‘Certainly out in primary care where people are using – so it would be technicians and things – you could certainly make a shoulder class or exercise class around such things… I think it would be useful’* [C5]

The use of the machine was coupled with the Patient App and in the early stages of the feasibility study there were occasions where the Patient App failed to work properly:

*‘The one downfall has been the reliability of the software for us as a clinician to use frequently and repeatedly to put up a full programme for a patient to use it as it was designed to’* [C4]

Despite these occasional issues, the Patient App and Physiotherapy Portal component of the MUJO System enhanced the clinician’s ability to deliver rehabilitation. Patients valued the ability to see visual feedback for the exercises they were completing on the iPad screen and it was useful to be able to record real time movement data to assist with clinical reasoning and measuring improvement:

*‘it does seem like a good idea from a training perspective, with patients being able to see what they are doing as a graphs such – on as far as the smoothness and coordination of their movements, that seemed to be a useful tool, but also being able to then review that with a patients when they are coming back’* [C3]

One of the major barriers to incorporating the MUJO System in clinical practice was its accessibility. The pilot site is a national specialist centre and patients often had to travel long distances to attend outpatient appointments. The usefulness of prioritising the use of the MUJO System when it was not available locally was questioned by all clinicians:

*‘It’s the repeatability. It’s them being able to learn an exercise here and then go home and, is always the way, a lot of that comes down to time. When I’ve got a short period of time to assess somebody and then treat them, I would rather treat them with the exercise they are going to do at home, rather than teach them something here and then say, and this is how we adapt it for you to carry on at home. It’s silly.’* [C3]

However, even when the patient could not use the machine for rehabilitation there was still an identified role for the MUJO System as an assessment tool:

*‘I could use it as an assessment tool to get specific markers to begin with. They go off and do their home exercises in any other ways and then come back and then I could reassess using it and see what happens numbers-wise with the [Patient] App’* [C2]

Patients who were local enough found being able to access the machine on a regular basis optimised their outcome:

*‘So I built it into my regime every day. I’d go to the machine first thing in the morning before anyone was using it, do about half an hour on it and then just crack on for the rest of the day. So I think it sped up my recovery considerably’* [P7]

And for one local patient who found the machine useful there would be a commitment to travel to use the device

*‘If I was far away from the MUJO machine, I don’t know what I would do, to be honest. I would probably travel to use it’* [P3]

### Capacity – Social structural resources available to patients and clinicians

The accessibility of the equipment for patients and clinicians was identified as the main barrier to using the device:

*‘I think in theory it’s a good piece of equipment, but it just needs – it’s not the equipment that needs to be developed so much, its more the availability of it’* [C3]

As a basic pre-requisite of using the device for rehabilitation both patients and clinicians felt that it was important for any healthcare professional to be suitably qualified and have knowledge surrounding the shoulder:

*‘Most physios should have a fairly basic understanding of what muscles are weak and dysfunctional. Therefore they shouldn’t need much support around where’s the weakness in rotators or in abductors and a combination of both … you would probably need some general understanding around shoulder muscle control to be able to use it appropriately’* [C2]

‘I wouldn’t want to get a programme from someone at the gym to show me how to use my shoulder. That’s because I am in rehab. Having been to the gym and having had shoulder surgery and they have suggested rowing, that was a personal trainer, that is not the level of person I would expect to be telling me – setting a programme for rehabilitation’

### Potential – Social cognitive resources available to patients and clinicians

Patients and clinicians felt the use of the Patient App enhanced the rehabilitation experience. It was frustrating when the Patient App did not work and on these occasions the physiotherapist would actively avoid using this aspect of the device:

*‘I’ve tended to log in and use it with a patient at that moment in time, but it’s not really collected any data. That’s because it’s frustrating that the syncing or whatever was supposed to be happening wasn’t occurring or there were recurrent problems. To do it in front of a patient is a little bit embarrassing so I stopped doing it. I just end up logging in as myself and getting the screen up for them, because that’s the useful bit’* [C5]

*‘the usability needs to be better for it to be able to be rolled out because I think if it was more user-friendly, the take up with the therapy team might be a bit better. Some people are put off if it’s difficult to use on one occasion’* [C5]

A physiotherapist reported how on one occasion a patient encountered difficulty with the app and their exercise regime was not recorded, negatively affecting their experience:

*‘They got quite frustrated, one that was quite competitive. She was like, I definitely did it and its saying I didn’t do anything. It said I did really badly’* [C7]

Some patients found the app useful and motivating. One patient, when asked about what appealed to them most about the MUJO System device responded:

*‘The [Patient] App. When it is turned on I can see like my first appointment I done say five percent rotation and then my next appointment I got up to thirteen so I’m slowly progressing’* [P10]

And the Patient App was enjoyable and seen to optimise compliance:

*‘Because the patients are so positive about it I’ve tended to find a way because one of the biggest challenges we face as therapists is patient adherence and compliance. Now we can give completely the right exercise to somebody but if they don’t enjoy it or find a way of being able to do them easily it’s not going to make much difference’* [C2]

Clinicians would avoid using the device if the patient was not going to be able to access it from home, usually because of the geographical distance they lived away from the machine. The Physiotherapists would actively use the machine if a patient could access it, and patients enjoyed the process of rehabilitation when they could use the Internal and External Devices and the Patient App:

*‘a good example now I’ve just recently got a patient who lives down the road, he is really keen to use it, I think the MUJO is really going to help him and I set him up on it’* [C2]

‘*I think the machine is brilliant. For me it’s helped me a lot because I can come and use it as and when I wish. I can come and use it as much or as little as I like, especially when I get days like today that I am having a bad day, so I can just do a little bit. I feel that its increased obviously the angle at which I can move my arm, I’ve gone from 30 to 60, although today I have gone back down to 30. So for me it has worked out quite well.’* [P3]

Both patients and clinicians recognised the potential of the machine to free up clinical time:

*‘I do think it reduces the amount of clinical episodes you need directly with the physio staff, so it basically releases your clinical time. So you can see more patients, because you are not reliable on so many sessions. So instead of maybe having, let’s say, six to ten sessions, you can easily half that, which is what happened to me’* [P7]

There was, however, an identified requirement to allocate time to check the exercises and compliance this time would need to be protected within the clinician’s diary. Replacing face to face contact with patients attending independently to use the device would have some cost implications, particularly in a healthcare system where payment is made for each face to face contact this was identified as a potential problem and barrier to implementation.

Clinicians had awareness that collective commitment was dependent on how they ‘sold’ the device to the patient. On occasions where clinicians were frustrated with the Patient App or Physiotherapy Portal not working this was likely to influence whether or not they would use the device with the patient.

*‘I think increasingly it’s all about how you sell it. I think the patients come to you and they want to get better and it’s very clear to them that this is what is going to make them better. So there has got to be belief. There has got to be belief on the clinicians part, there’s got to be belief on the patients part, that the intervention, whatever it is, is going to improve them’* [C2]

### Contribution – What patients and clinicians do to implement the MUJO system in clinical practice?

Clinicians reported they would actively try to empower patients to use the device as part of their normal rehabilitation to promote patient compliance and release clinician time and in these situations MUJO was effective. On occasions when patients were not in a position to use the device on an ongoing basis the clinicians would not actively use the MUJO System for rehabilitation with their patients.

Through the use of the machine over the feasibility study period, the physiotherapists learnt how best to use the device to support their patients and grew increasingly aware of the capabilities of the device and used the Internal and External Devices for specific rotator cuff muscle strengthening exercises and found this was preferable to other available machines:

*‘I think as far as gym equipment is concerned, they don’t have pieces of equipment that are so cuff specific. Yes we know we are not completely isolating the cuff, but we are being more specific than you are with a lot of gym equipment. The majority of gym equipment is unsupported, so you are automatically incorporating your bigger mobilisers, deltoid, lats, pecs, with a lot of the exercises you do in gyms’* [C3]

In certain circumstances, such as kinetic chain rehabilitation, progressing the patients shoulder stability with the Internal and External Devices was more limited:

*‘We see a lot of very challenging multidirectional instability which is quite a select cohort. They come with global issues, so kinetic chain, scapula control problems, not just glenohumeral’* [C2]

*‘The only thing is, obviously with progressions, what we try and do is reduce the support. So from a strength component, MUJO is good. But if we want to reduce the stability, we can’t really do that’* [C4]

For those patients who could access the device daily, particularly those who were attending residential rehabilitation, the clinicians would encourage the patients to use the MUJO System:

*‘I’ve not used for all outpatients, but for the rehab patients it worked well. So I could set them up and they would come and do it themselves without the need for me to be there to show them how to do it or monitor them or give them feedback’* [C7]

For outpatients however, there was not enough time to manage the patient and set up the machine and the Physiotherapy Portal and Patient App so clinicians were less likely to use it, particularly if the patient was unable to access the device:

*‘In the 30 minute consultation, you could probably do the setting up with the patient if that – the thing is if that’s the only thing you wanted to do. So if you wanted to do other things in your session like check other exercises or assess – I don’t know if you – if that was the only plan to just set up the MUJO then you would have time I think in a session to do that’* [C7]

## Discussion

In this study Normalisation Process Theory [14] has been used to focus attention towards the factors that would lead to the MUJO System being accepted and normalised within a healthcare setting.

Using the MUJO System introduced a new way of working for patients and clinicians as the physiotherapists were required to set up an account for patients on the Physiotherapy Portal in order for patients to access the Patient App. The main factor for technology to be implemented in healthcare is acceptability [16]. In situations where the use of the Physiotherapy Portal and Patient App interfered with the normal working practices of the physiotherapist clinicians would take shortcuts to avoid using it, particularly when the Physiotherapy Portal and Patient App was introduced in the early stages of its development. The uptake of the MUJO System was threatened by the amount of time it took to set up the Devices, Physiotherapy Portal and Patient App and this may be challenging in departments where appointment time slots are as low as twenty minutes.

Despite some reported initial challenges of using the Patient App however, it was seen as a useful adjunct to normal care. Adherence to exercise involves a number of factors, including how often the patient performs the exercises, whether the quantity of the exercise performed is sufficient to provide a therapeutic benefit, and how long the patient continues to perform these exercises [17]. The ability to monitor patients from a distance via an online portal was seen to promote patient compliance and adherence. A device with a connected Patient App such as the MUJO System may facilitate adherence through its reminder service for users to complete their exercise prescriptions, an initiative that has been shown to effectively reduce Did Not Attend (DNA) rates at hospital appointments [18].

In comparison to home based exercises using exercise bands or hand weights to exercise the rotator cuff, clinicians reported the usefulness of the Internal and External Devices in being able to perform similar exercises in a way that targeted specific muscles but with more objective measurement and feedback [4]. However, clinicians felt the device was limited when more functional based exercises which required improvements in global posture and or lower limb strength were required.

For some patients, the Internal and External Devices made them feel more secure which may reduce pain related fear of movement [19, 20] and could be a useful tool to get patients confident in moving again. The majority of shoulder problems are managed in primary care by general practitioners and physiotherapists and national guidance advocates this care should include patient education and exercise prior to considering more invasive treatments [21]. Clinicians reported that easy access and wider availability of the MUJO System could further enhance rehabilitation in the community as well as reduce the burden on local services. For the MUJO System to be a routine component of shoulder management in primary care further clinical trials to evaluate its clinical effectiveness and socioeconomic benefits would be required prior to consideration of resources and changes to infrastructure shifting from hospital to more community based settings.

The largest reported study of telehealth and telecare, the Whole Systems Demonstrator study [7] reported that if delivered properly, telehealth can reduce the demand on health services and in this study we found that the use of the MUJO System had the potential to reduce the requirement for face to face sessions with the physiotherapists when patients could access the devices and further quantitative studies are required to investigate this further.

In this study both patients and clinicians stressed the importance of having a good understanding of the shoulder complex when setting rehabilitation programmes and the structures involved and mechanisms contributing towards the pathology [22]. It has been reported elsewhere that professional knowledge and training was a barrier to people with disabilities and that several fitness and recreation professionals commented that fitness facility staff such as personal trainers are not knowledgeable about these disabilities [23]. When identifying the environment for housing the Internal and External Devices in the community the information and training requirements of staff in the host site would be an important consideration to facilitate patient compliance. In some cases patients may prefer conventional shoulder rehabilitation rather than from a distance [24] and this is in accord with a study where patients stressed that remote consultations should be a supplement to care and not a replacement for any face to face contact with their healthcare professionals [25]. In a study looking at exercise adherence and dropout [26] non adherers were more afraid to access support and adherers felt good about themselves for having support. When moving rehabilitation and care out of a hospital environment and empowering patients to continue this in a community setting it is clear that there will be ongoing support requirements for such an intervention to succeed and indeed in our study patients stated would still want to access the expertise of the physiotherapist throughout their rehabilitation as required.

The results of the study should be interpreted in light of its potential limitations. Clinicians and patients who were already using the MUJO were invited to participate. All clinicians involved had received training in the use of the MUJO system and the funding from the project grant enabled the equipment to be loaned to the host institution. All patients had used the devices and were identified by their clinicians. Interviewing patients who chose not to use the MUJO may have provided information on the reasons why the devices were not acceptable and reduced potential biased reporting.

## Conclusions

Both clinicians and patients perceived the MUJO system had the following advantages:

1. It helped to target particular shoulder muscles
2. Enhanced patient motivation and compliance
3. Provided some quantifiable measure of performance
4. The machines were easy to set up with patients
5. Appeared to be clinically effective

However the MUJO system also posed some perceived disadvantages:

1. As an adjunct to current treatment for some clinicians it added consultation time
2. Poor software reliability reduced motivation for clinicians to use
3. Exercises were not function specific
4. It was only useful as a rehabilitation tool if the patient lived close enough to the devices to use them

For digital technology such as this to be accepted more widely systems have to be more accessible for patients to use remotely and data capturing has to be integrated into current electronic patient records so as not add to clinician time. Robust clinical trials would be required to demonstrate the MUJO system has a significant clinical benefit and cost benefit over current conventional methods of delivering shoulder rehabilitation before it could be more widely adopted by the NHS.

For the MUJO System to be taken up in clinical practice it needs to be workable within the context of the treatment session and not interfere with the clinician’s workload. Both patients and clinicians made it clear that the healthcare professionals setting the rehabilitation plan must have a thorough understanding of the shoulder complex and pathology and be knowledgeable about exercise prescription.

For those patients who lived locally to the hospital and could access the device the clinicians would encourage the patients to use the system in their rehabilitation. Clinicians in this study found the use of the MUJO System was not acceptable for rehabilitation if the patient was unable to access the device in the community. In situations where technical issues arose with the Patient App and Portal this served as a barrier to implementation. Further quantitative research is required to explore whether the MUJO can reduce the demand on the physiotherapist through using up less 1:1 treatment sessions.

## Additional files

Additional file 1: Appendix 1. An interview schedule. An interview schedule of questions the interviewer asked patients and clinicians. (DOCX 28 kb)

Additional file 2: Patient demographics. This is a table to demonstrate the patient demographics in the study. (DOC 36 kb)

## Abbreviations

Cuff: Rotator cuff
Internal and External Devices: Exercise machines targeting the internal rotators and lateral rotators of the shoulder respectively
MUJO System: Multiple Joint System [6]
Patient App: Application component of MUJO System
