# Supplementary material for: The use of assistive technology in shoulder exercise rehabilitation – a qualitative study of acceptability within a pilot project
Source: BMC Musculoskelet Disord. 2018 May 2;19:133. doi: 10.1186/s12891-018-2042-6 (PMC5930518; doi:10.1186/s12891-018-2042-6)
Supplement: Supplementary file 1 — Appendix 1. An interview schedule. An interview schedule of questions the interviewer asked patients and clinicians. (DOCX 28 kb) [file 12891_2018_2042_MOESM1_ESM.docx]

**Appendix 1 – Semi Structured Interview Guide**

**NPT constructs and practical research questions: emergent expressions of agency.**

|  | Construct | Sub Construct | MuJo question | |
| --- | --- | --- | --- | --- |
|  |  |  | Patients | Clinicians |
| Emergent expressions of agency | Capability | Workability | - What was your experience of using the MuJo machine & app like? - What was different about using MuJo compared to normal rehab? - What was the most challenging thing about using MuJo & app? - What was the easiest thing about using Mujo & app? - Were there any skills you feel you needed to use MuJo & app? | - What was your experience of using the MuJo machine & app like? - What was different about using MuJo compared to normal rehab? - What was the most challenging thing about using MuJo & app? - What was the easiest thing about using Mujo & app? - Were there any skills you feel you needed to use MuJo & app? |
|  |  | Integration | - Do you feel the same level of confidence with rehab using MuJo? - What could be done to improve your confidence with using MuJo? - Was there anything difficult about doing shoulder exercises using MuJo? - Is there anything MuJo can’t do compared to normal rehab? - Is MuJo fit for purpose for shoulder rehab? | - Do you feel the same level of confidence with rehab using MuJo? - What could be done to improve your confidence with using MuJo? - Was there anything difficult about teaching shoulder exercises using MuJo? - Is there anything MuJo can’t do compared to normal rehab? - Is MuJo fit for purpose for shoulder rehab? |
|  | Contribution | Coherence | - Had you ever used MuJo (or similar) before? - What were your expectations of using MuJo? - What do you think the benefits are of using MuJo rather than normal? - How much support did you need to understand the benefits of MuJo before making your decision to use it? - How valuable do you think MuJo will be in the future? - Do you think you used the MuJo effectively? | - Had you ever used MuJo (or similar) before? - What were your expectations of using MuJo? - What do you think the benefits are of using MuJo rather than normal? - How much support did you need to understand the benefits of MuJo before making your decision to use it? - How valuable do you think MuJo will be in the future? - Do you think you used the MuJo effectively? |
|  |  | Cognitive Participation | - How much did your clinician influence your decision to use MuJo? - What appealed to you about MuJo? - What is the best part of MuJo? - Why didn’t you choose not to use MuJo? - What would be important for us to do, to promote patient engagement with MuJo in the future? - If a friend asked for your advice, about whether to use MuJo or not, what would you say? | - How much persuasion did you need to use MuJo? - What appealed to you about MuJo? - What is the best part of MuJo? - Why didn’t you choose not to use MuJo? - What would be important for us to do, to promote clinician engagement with MuJo in the future? - If a colleague asked for your advice, about whether to use MuJo or not, what would you say? |
|  |  | Collective Action | - What support was offered to you after you chose use MuJo? - What support would you recommend we provide to other patients who might want to use MuJo? - Do you think we need to offer training to patients, if so, what? | - What support was offered to you after you chose use MuJo? - What support would you recommend we provide to other clinicians who might want to use MuJo? - Do you think we need to offer training to patients and clinicians, if so, what? |
|  |  | Reflexive Monitoring | - What would you say are the benefits of using MuJo - What are the problems of using MuJo? - Do you think the RNOH offering a MuJo service is worthwhile? - How might we improve the MuJo service? | - What would you say are the benefits of using MuJo - What are the problems of using MuJo? - Do you think the RNOH offering a MuJo service is worthwhile? - How might we improve the MuJo service? |

**NPT constructs and practical research questions: dynamic elements of context.**

|  | Construct | Sub Construct |  |  |
| --- | --- | --- | --- | --- |
|  |  |  | Patients | Clinicians |
| Dynamic elements of context | Capacity | Material Resources | - What equipment did you use for use of the app? - Did you need any additional equipment? - What was the sound and picture quality like? - Is there any additional equipment you feel is necessary? | - Do you have the equipment available for you to use the MuJo and app? - Did you need any additional equipment? - What was the sound and picture quality like? - Is there any additional equipment you feel is necessary? |
|  |  | Informational  Resources | - Did you feel you understood how to use MuJo? - Was there any extra information support you required to use it? - What information support would be useful for patients in the future? | - Did you feel you understood how to use MuJo? - Was there any extra information support you required to use it? - What information support would be useful for clincians in the future? |
|  |  | Social Norms | - You know how normal rehab is, how did it feel using MuJo? - How did using MuJo alter your relationship with the clinician? - How would you feel using MuJo in the community? | - You know how normal rehab is, how did it feel using MuJo? - How did using MuJo alter your relationship with the patient? - How would you feel about patients using MuJo in the community? |
|  |  | Social Roles | - How did you find the interactions were with your clinician using MuJo? - Would this be different in a normal rehab scenario? - How would you describe the typical ‘Patient clinician relationship’ - If we think of the typical ‘clinician patient relationship’ how does MuJo affect this? | - How did you find the interactions were with your patient using MuJo? - Would this be different in a normal rehab scenario? - How would you describe the typical ‘Patient clinician relationship’ - If we think of the typical ‘clinician patient relationship’ how does MuJo affect this? |
|  | Potential | Individual Intentions | - What was your motivation to participate with the MuJo? | - What was your motivation to participate with the MuJo? |
|  |  | Shared commitments | - Using MuJo was new for your therapists also, what problems did you have together, and what did you and the clinician have to do to make the consultation work? - Do you think your clinician ‘bought in’ to MuJo - Would you use MuJO with your clinician again, or have ‘normal’ rehab? | - Using MuJo was new for your patient also, what problems did you have together, and what did you and the patient have to do to make the consultation work? - Do you think your patient ‘bought in’ to MuJo - Would you use MuJO with your patient again, or have ‘normal’ rehab?? |
