# Supplementary material for: The use of assistive technology in shoulder exercise rehabilitation – a qualitative study of acceptability within a pilot project
Source: BMC Musculoskelet Disord. 2018 May 2;19:133. doi: 10.1186/s12891-018-2042-6 (PMC5930518; doi:10.1186/s12891-018-2042-6)
Supplement: Supplementary file 2 — Patient demographics. This is a table to demonstrate the patient demographics in the study. (DOC 36 kb) [file 12891_2018_2042_MOESM2_ESM.doc]

**Supplementary Information**

| Participant No | Age at Presentation / Gender | Duration of Symptoms | Diagnosis | In or out patient Rx | Surgery |
| --- | --- | --- | --- | --- | --- |
| P1 | 31-40 age bracket | 2 years | Chronic bilateral atraumatic instability with secondary impingement | In patient | none |
| P2 | 31-40 age bracket | 6 years | Right ACJt stabilisation | Outpatient post op | Right ACJt stabilisation Jan 2016 |
| P3 | 51-60 age bracket | RTA 2008 referred 2015  7 year Hx | Chronic Left shoulder pain | Out patient | None |
| P4 | 31-40 age bracket | 1 year Hx | Multi-directional instability | Out patient | Arthroscopic posterior bankart repair Dec 2013  Revision stabilisation , biceps tenotomy and SAD May 2015 |
| P5 | 31-40 age bracket | Since teens  Over 10 years | Atraumatic chronic instability and pain | Out patient | Arthroscopic Stabilisation Oct 2015 |
| P6 | 21-30 age bracket | 7 year Hx | Left recurrent shoulder instability | In patient & out patient | Arthroscopic stabilisation 2015 |
| P7 | 41-50 age bracket | 3 months | #/dislocation right shoulder with partial denervation of deltoid | Out patient | None |
| P8 | 41-50 age bracket | 10 year Hx | Chronic Right shoulder pain | Out patient | Revision ACjt stabilisation June 2015 |
| P9 | 21-30 age bracket | 6 year Hx | Left recurrent postero-inferior instability | In patient rehab | Previous stabilisation 2011 |
| P10 | 18-20 age bracket | 2 years | Left Posterior shoulder instability atraumatic | In -patient | Diagnostic arthroscopy Nov 2015 |
